# Optimal Reaction Coordinate as a Biomarker for the Dynamics of Recovery from Kidney Transplant

**DOI:** 10.1371/journal.pcbi.1003685

**Published:** 2014-06-26

**Authors:** Sergei V. Krivov, Hayley Fenton, Paul J. Goldsmith, Rajendra K. Prasad, Julie Fisher, Emanuele Paci

**Affiliations:** 1 School of Molecular and Cellular Biology, University of Leeds, Leeds, United Kingdom; 2 Astbury Centre for Structural Molecular Biology, University of Leeds, Leeds, United Kingdom; 3 School of Chemistry, University of Leeds, Leeds, United Kingdom; 4 Hepatopancreatobiliary Transplant Unit, St. James's University Hospital, Leeds, United Kingdom; National Research Council of Canada, Canada

## Abstract

The evolution of disease or the progress of recovery of a patient is a complex process, which depends on many factors. A quantitative description of this process in real-time by a single, clinically measurable parameter (biomarker) would be helpful for early, informed and targeted treatment. Organ transplantation is an eminent case in which the evolution of the post-operative clinical condition is highly dependent on the individual case. The quality of management and monitoring of patients after kidney transplant often determines the long-term outcome of the graft. Using NMR spectra of blood samples, taken at different time points from just before to a week after surgery, we have shown that a biomarker can be found that quantitatively monitors the evolution of a clinical condition. We demonstrate that this is possible if the dynamics of the process is considered explicitly: the biomarker is defined and determined as an optimal reaction coordinate that provides a quantitatively accurate description of the stochastic recovery dynamics. The method, originally developed for the analysis of protein folding dynamics, is rigorous, robust and general, i.e., it can be applied in principle to analyze any type of biological dynamics. Such predictive biomarkers will promote improvement of long-term graft survival after renal transplantation, and have potentially unlimited applications as diagnostic tools.

## Author Summary

The evolution of disease or the progress of recovery of a patient is usually monitored by collecting physical parameters, which may be simply the body temperature for a common cold or properties of tissue samples for e.g., cancer. Most often clinical decisions are taken based on the current value or because of a sizable change of a relevant parameter. As more advanced diagnostic tools become available, and huge numbers of parameters can be collected at short, frequent time intervals, two related questions arise. The first is, which of the parameters provides relevant information on the progress of disease or recovery as opposed to noise? Is there more information that can be obtained from the history of the evolution of such parameters? Here we propose a novel approach that leads, for the specific case of recovery from kidney transplant, to a positive answer.

## Introduction

The responses of an individual to an infection, to pharmacological treatment or to surgery are examples of time-dependent stochastic processes characterized by complex dynamics. An increasing amount of time-resolved data is available reporting on the unique chemical fingerprints that specific cellular processes leave behind [1], [2]. Metabolites, such as those found in blood or urine, contain in principle a comprehensive picture (referred to as the metabolome) of the evolution of a patients condition. While such a picture is very complex and generally not insightful, the time evolution of the metabolome of a patient contains crucial information. Conventionally, a biomarker is sought by comparing differences in the metabolic profiles between two states (e.g. healthy and pathological) using unsupervised methods (such as principal component analysis, PCA [3]) or supervised methods (e.g., orthogonal projections to latent structures, OPLS [4] and related [5]). However, if one is interested in time-related changes to the metabolic profile, which are relevant to the pathological state, for example in the monitoring of disease progression or defining surrogate end points, the problem becomes more complex. A number of other methods, sometimes borrowed from other disciplines, have been proposed for analysis of time-resolved metabolomic data [2]; they rely in general on previous knowledge, either of the identity of the relevant metabolites and/or the functional form of the time dependence of their concentrations. When the underlying biochemical mechanism is itself unknown, such methods are obviously not useful.

Disease dynamics, according to the systems biology point of view, is more accurately described as dynamics of highly entangled molecular networks, with disease being an emerging property of the networks [6]. Adopting this view, we seek a biomarker, which is a descriptor (function) of the networks states, rather than of a few molecules. We assume that disease dynamics is a Markov (memory-less) stochastic process, in which future behavior is completely specified (in a probabilistic sense), by the current state of an organism, e.g., the complex of genome, proteome, metabolome, epigenome, age, environment, and whatever additional information may be required (hereafter the “configuration space”). Illustrative and enlightening is a recent study [1] where a combination of genomic, transcriptomic, proteomic, metabolomic and autoantibody profiles from a single individual was followed for over a 14 month period. The analysis uncovered extensive dynamics changes in diverse molecular components and biological pathways across healthy and disease states. In the case where the dynamics is stochastic rather than deterministic, a single observed trajectory is not sufficient for a complete description. In principle, a Markov state model that gives a complete description of the process can be constructed by observing various realizations of the disease, and computing the transition probabilities between all states. The model can be used to predict the properties of interest, for example, the probability of a given outcome (e.g., full recovery) after a certain time given an initial state. Such a straightforward approach cannot be realized in practice. The amount of information necessary to define exactly the state of an organism is huge and difficult to identify; the statistics necessary to construct the Markov state model grows exponentially with the dimensionality of the configuration space. Moreover, to be useful for practitioners (e.g., for diagnostic purposes), the description of the disease dynamics needs to be simplified. This can be done by introducing one or a few variables (hereafter the “reaction coordinate”) that describe the properties of interest. Ideally, such a simplified description should be as predictive as the Markov state model previously described, i.e., the probability of a particular clinical outcome calculated from the value of the variable should closely approximate that computed based on the full Markov state model. If such properties are satisfied we call the variable an “optimal reaction coordinate”.

### Determination of the optimal reaction coordinate

Here we present a general framework that allows us to determine such an optimal coordinate or biomarker from longitudinal cohort studies directly, without constructing the Markov state model. The method was originally developed to describe complex dynamics of protein folding [7]–[9]. Briefly, a putative functional form of the reaction coordinate is assumed, for example, a linear combination of features (here the metabolome 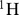 NMR spectra) that could describe the process. The approach is invariant to the choice of the functional form and the set of observables, provided they contain all the essential information about the dynamics of the process. The coordinate is optimized (trained) on a sample of trajectories, i.e., realizations of a complex multidimensional dynamical process. This is achieved by choosing the coordinate (e.g., the coefficients of the linear combination) such that the cut based free energy profile associated with the coordinate is the highest [7], [10]. Namely, given an ensemble of N trajectories 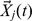 (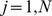) and a reaction coordinate functional form 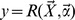 an ensemble of reaction coordinate trajectories is constructed by projecting the multidimensional trajectories onto the reaction coordinate 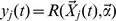 (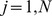). The optimal reaction coordinate is found by optimizing the parameters 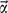 so that the cut-based free energy profile 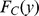 [7], [8] is maximal. 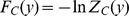, where the partition function 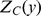 equals half number of transitions (crossings) by the reaction coordinate time-series through point *y*; here and below we set 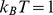. The CFEP (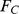) unlike the conventional histogram based free energy profile (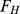) is invariant to reaction coordinate rescaling, insensitive to statistical noise and capable of detecting sub-diffusion. Together they determine the coordinate dependent diffusion coefficient 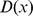 and thus completely specify diffusive dynamics [9]. One can maximize instead the generalized cut based free energy profile 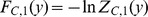 where the partition function 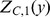 takes into account each transition through point *y* with weight equal to the transition distance; for a Gaussian distribution of steps (i.e., diffusive dynamics) the two optimality criteria are equivalent [10]. If the reaction coordinate is a weighted sum of basis functions 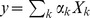, as used here, the optimal values of the parameters (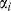 that maximize 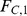 can be found analytically [10]).

In supervised optimization a coordinate that accurately describes the dynamics of transition between two given end states (e.g., healthy and disease) is determined. Incidentally, the coordinate is the probability of full recovery, i.e., of ending up in the “healthy” state rather than the “disease” state starting from a current state. It is known as committor or folding probability in protein folding studies [10], [11]. If the two end states are separated by the highest free energy barrier, the transition between them corresponds to the slowest relaxation mode, and an eigenvector, corresponding to the slowest mode is an optimal reaction coordinate [10], [12]. This coordinate can be determined in an unsupervised way without explicit definition of end states.

## Results/Discussion

The method outlined above (details given in Materials and Methods) has been used to analyze the evolution of 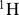 NMR spectra of the erythrocyte extracts of blood from 18 patients undergoing kidney transplantation; for each patient up to nine samples were taken before surgery and daily up to one week after. The spectra were normalized to the total sum of the spectral intensities and then coarse-grained with a bin size of 0.32 ppm. The average intensity within each bin was logarithmically transformed as 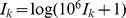. The reaction coordinate was taken as a linear sum of the transformed average bin intensities: 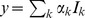. The method to determine the optimal reaction coordinate is robust: it was repeated with different transformation (e.g., 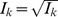) or without transformation, with different bin sizes, in a supervised way, all leading to virtually identical results. Note however, as discussed below, a significant decrease of the bin size, e.g., to 0.1 ppm, while leading to a slightly better description results in over-fitting.

Independently of the NMR data, patients have been divided in three classes based on a clinical assessment of the patients into “primary function” (PF), “delayed graft function” (DGF) and “acute rejection” (AR) with and without primary function. Primary function was defined as immediate recovery of renal function following surgery. Delayed graft function was defined as the need for dialysis in the first week following transplantation.

**Figure 1:**
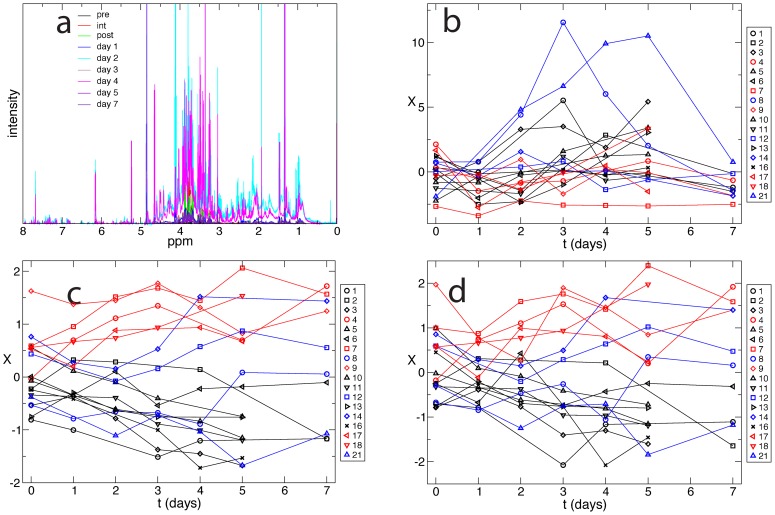
Unsupervised optimization. a) NMR spectra for blood extracts of a single patient, collected over nine time points. b) Patients trajectories projected on the first principal component do not show any feature enabling us to separate the trajectories according to the clinical assessment of the patients. The color indicates the final clinical classification of the patient: primary function patients are shown in black, delayed graft function in red, and acute rejection in blue. c) Patients trajectories projected on the optimal reaction coordinate. d) Leave-one-out cross-validation: every trajectory is projected on the optimal reaction coordinate constructed without that specific trajectory.

The spectra for a single patient for nine different time points (a “trajectory”), are shown in Fig. 1a. The trajectory for each patient projected onto the first principal component (Fig. 1b) shows individual variability but no separation between different classes of patients, and no relevant information on the evolution of their clinical conditions during the period in which the samples were taken. The same is true if the trajectory is projected onto the first few principal components.

Fig. 1c shows patients trajectories projected onto the optimal reaction coordinate determined in the unsupervised way (the second eigenvector). Trajectories categorized as PF are characterized by an evolution towards negative values of the coordinate; those in the DGF cohort evolve towards positive values of the coordinate. The time evolution of the AR patients cannot be discerned within the timescale of the data collection. Importantly, the clinical group to which each patient could be ascribed to is apparent from about the second day after surgery, earlier than any other clinical indicator, including invasive, though gold-standard, biopsy. Note that separation between PF and DGF patients cannot be due to dialysis, since dialysis was never performed in the first two days after surgery.

The difference between the results of PCA (Fig. 1b) and of the approach proposed here (Fig. 1c) can be understood as follows. PCA (and other algorithms [10]) perform dimensionality reduction with a focus on representation of the properties of the configuration space. In particular, the PCA maximizes the variability of configurations along the principal components. The dynamical information contained in the temporal sequence of congurations (trajectory) is ignored. By explicitly considering the dynamics, our approach performs dimensionality reduction while preserving and exploiting the dynamics of the process [10], [13].

To demonstrate that the results are not affected by over fitting (even though the analysis is unsupervised) the leave-one-out cross-validation procedure was performed. In Fig. 1d every trajectory is projected on the optimal reaction coordinate constructed without the trajectory. All the trajectories are in good agreement with those in Fig. 1c, and the prediction on the future evolution of each trajectory (i.e., the fate of each patient) is identical. This confirms that the constructed coordinate is robust and that the biomarker can be used to follow up the evolution of the condition of a new patient.

The leave-one-out cross-validation was also instrumental in the choice of the bin size. Decreasing the bin size increases the number of parameters and thus the flexibility of the coordinate; while this leads to a slightly better separation, the cross-validation test fails, a clear consequence of over fitting. Note that, in principle, the possibility of over fitting, as well as the optimality of the coordinate, could be established by comparing the cut profiles computed with different time intervals [8], [14] or with other methods [15]–[17]. Alternatively, one may optimize with an over fitting penalty [8], eliminating the need in the manual choice of the bin size. Here we did not use such an approach, because the trajectories are too short to be sampled with larger intervals.

**Figure 2:**
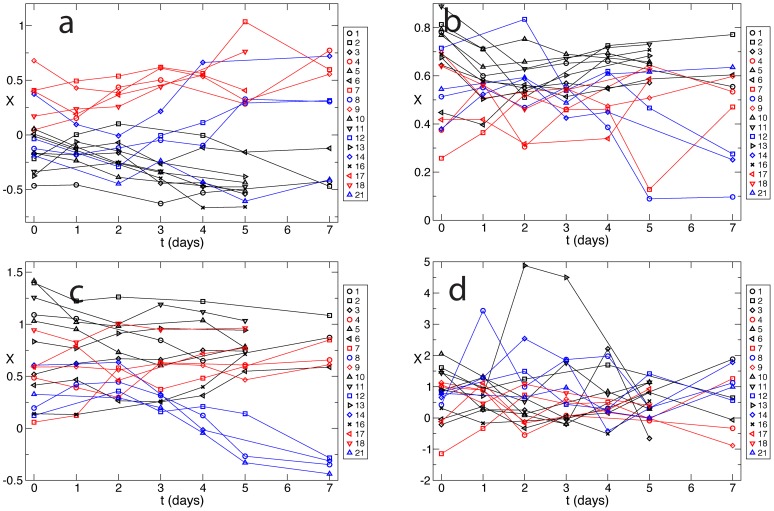
Supervised optimisation. **a)** PF versus AR and DGF with binning interval of 0.32 ppm: the results are similar to the unsupervised analysis Fig. 1C. **b)** AR versus PF and DGF with binning interval of 0.32 ppm: no clear separation between the classes. **c)** AR versus PF and DGF with binning interval of 0.1 ppm: AR is separated from PF and DGF. **d)** leave one out cross validation of panel **c**: small binning interval of 0.1 ppm leads to overfitting.

While the second eigenvector describes the transitions dynamics over the highest barrier, it is possible that the third eigenvector, which describes the second slowest process, would separate AR cases from PF and/or DGF cases. However, this is not the case. Among the many possible reasons why AR cases cannot be discriminated from the other cases, one could be the insufficient flexibility of the reaction coordinate due to the small number of parameters. Increasing the number of parameters while avoiding over fitting would require more trajectories (i.e., more patients) than those available. To illustrate this fact we performed a supervised analysis aimed at separating AR from PF and/or DGF trajectories. Fig. 2a shows supervised optimization where PF trajectories were terminated at −1 and AR and DGF trajectories at 1. The results are virtually identical to those obtained by unsupervised optimization reported in Fig. 1C. An attempt to separate AR from PF and DGF with 0.32 ppm binning interval did not produce meaningful results (Fig. 2b). Decrease of the size of the binning interval to 0.1 ppm resulted in a more flexible reaction coordinate, so that the supervised separation of AR from PF and DGF become possible Fig. 2c. However, results of leave-one-out cross-validation, shown in Fig. 2d illustrate that such small binning interval leads to over fitting.

**Figure 3:**
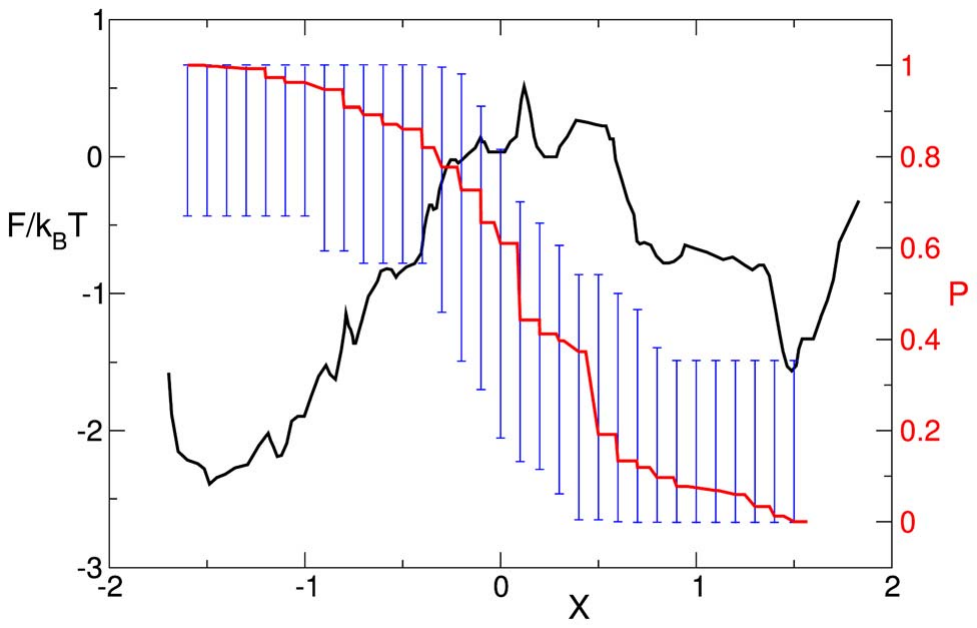
The disease dynamics is described as diffusion on the free energy landscape (black). The left and right basins correspond to PF and DGF+AR states, respectively. Probability of the successful outcome from current conditions P(x) computed from diffusion on the free energy landscape (red line) and directly from the trajectories (the blue vertical bar represents 95% confidence interval on the estimation).

Having determined an optimal coordinate, the dynamics of disease as a whole can be described as diffusion on the free energy profile (Fig. 3) along the optimal coordinate. The latter has been rescaled so that the diffusion coefficient equals unity. Two basins (attractors) naturally emerge, one identifying the PF condition and one for DGF and AR conditions together. Starting from the top of the profile, a patients trajectory can fall either on the left (PF) or the right (DGF+AR) basin. Fig. 3 shows the probability of full recovery, i.e., of reaching the left basin (PF) before reaching the right one (DGF+AR) starting from any value of the coordinate (any condition before or after surgery) computed by assuming diffusive dynamics on the free energy profile and directly from the trajectories. Their agreement in combination with the cross-validation (Fig. 1d) demonstrates the possibility to predict the clinical outcome for patients not previously considered, meaning that the optimal coordinate found is a good biomarker.

**Figure 4:**
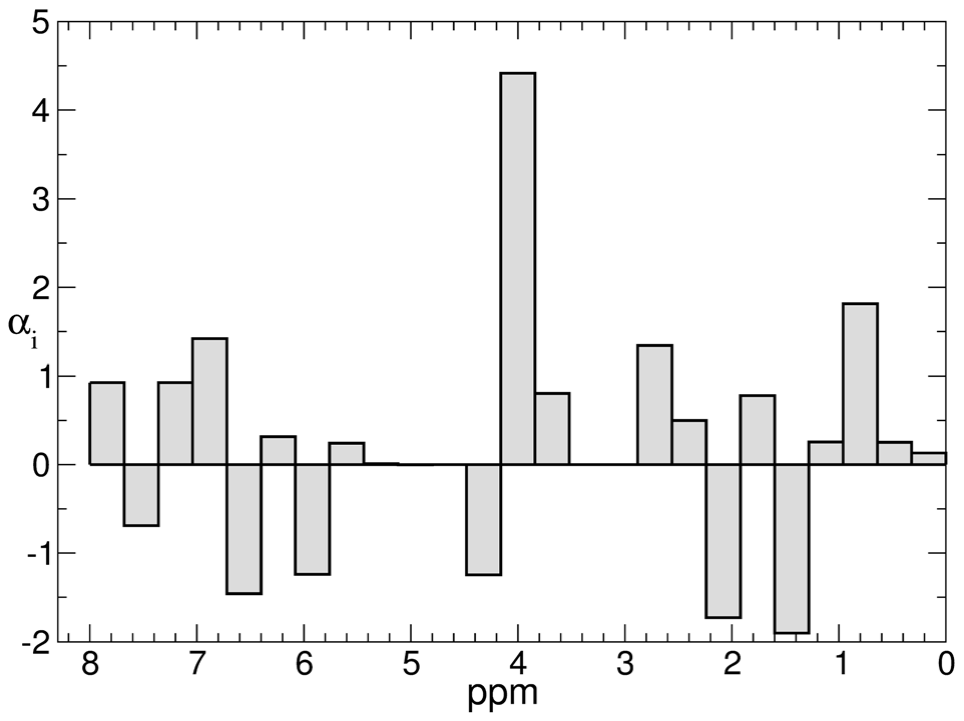
Linear coefficients of the optimal reaction coordinate 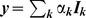, where *I_k_* is the logarithm of the intensity of NMR spectra in bin k.

The linear coefficients of the optimal reaction coordinate (Fig. 4) illustrate that the whole spectrum (metabolome) is important to determine the optimal coordinate that provides a fine-grained quantitatively accurate description of the dynamics. Indeed, the largest weight is associated with the bin ranging from 3.84 to 4.16 ppm; this includes signals for creatinine. Creatinine is currently used as a clinical marker in the assessment of renal function and to monitor post-transplant recovery [18]; the trend in the serum creatinine concentrations is more informative than absolute values, but it is by no means a specific marker. Other signals, e.g., at ca. 1 ppm/2 ppm and 1.4 ppm correspond to lipid and lactate respectively; the signal for the CH of lactate is at 4.2 ppm. We note that while the identity of each of the metabolites is undoubtedly interesting *per se*, their identities are not required for the usage of the optimal coordinate for diagnostic purposes as a biomarker.

In order to describe the specificity and sensitivity of a proposed biomarker one commonly uses the ROC analysis, which describes the trade-off between false positives and false negatives [19]. The proposed approach differs from the conventional ones (e.g., the PCA) by assuming that the disease dynamics is stochastic, which makes an application of such analysis not straightforward. This can be understood by considering a set of patients and a vector of measurable characteristics (e.g., the NMR spectra) for 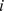-th patient denoted as 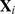. Conventionally, one assumes that the true state of a patient, denoted by 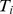, can be unambiguously mapped (by a “gold standard” test) onto two classes 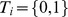, e.g., healthy and diseased. And that this mapping is deterministic, i.e., that if 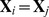, then 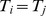. A biomarker is then a binary function 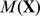 from 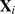 onto 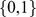 which best approximates 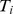. Cases where 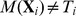 correspond to the two possible types of errors - false positive and false negative. The former, for example, corresponds to the fraction of cases where 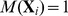, while 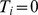. Since 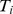 are determined by a “gold standard” test, the biomarker function is assumed to be the sole source of errors.

The proposed approach assumes that the current state of a patient 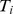 is related to the two terminal states 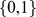, where the patient will end up eventually, only probabilistically. Identical patients with identical conditions undergoing identical treatment (i.e., 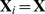) will end up in different terminal states 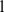 and 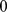 with probabilities of 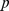 and 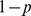, respectively. Thus knowing the terminal state does not allow one to determine the true value of the current state 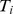, i.e., a “gold standard” test and stochastic dynamics are incompatible. Correspondingly, the purpose of a biomarker is not to approximate the terminal states but rather to approximate the probability 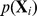 of ending up in one of the two end states.

A way to asses the accuracy of such a biomarker is to judge how accurately it reproduces that probability, e.g., Fig. 3. The classification process is analogous to the Bernoulli trial of a binary random variable that accepts 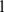 with probability 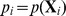. To determine the probability 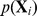 of ending up in the terminal state 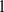, starting from 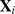, one needs to repeat “the experiment” a number of times starting from the same conditions 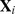 (in particular, the same patient) and count the fraction of events ending up in 1. Such a direct approach is clearly unrealistic. An alternative is to combine the states 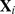 with similar 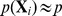 and determine 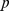 from such an ensemble of states, as have been done in Fig. 3.

### Conclusion

We have shown that the dynamics of recovery from kidney transplant can be quantitatively described as diffusion on a free energy profile, which is a function of a measurable biomarker. Such a biomarker can be determined in an (un)supervised way from longitudinal cohort studies (patient trajectories), which is optimal in the sense that it is able to discriminate where each patient is on a free energy profile. In particular, the probability of rapid recovery (primary function) can be used to devise optimal treatment. Such an approach is general and can be useful to develop optimal biomarkers for diseases that develop slowly and in a complicated way depending on many factors, or unknown unknowns, such as aging [20], cancer [21], [22] and psychological disorders [23].

## Materials and Methods

### Ethics statement

Approval was given by the regional ethics approval committee approval number REC Ref: 07/H1306/129

### The cut-based free energy profiles

The partition function of the cut-based free energy profile 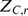 at point 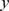 equals half the sum of the distances of those trajectory steps that go through point *y* [14]. More precisely,
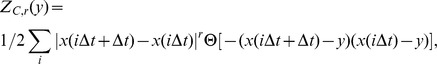
where 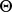
(*x*) is the Heaviside step function and *x*(*i*Δ*t*) is the reaction coordinate time series sampled with time interval Δ*t*. The cut free energy profile is defined as 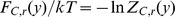 and 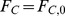; here we assume that 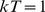.

The optimal coordinate is defined as the one with the highest cut profiles (lowest partition function). The justification of the optimization criteria can be summarized as follows (for the details see the cited references). It can be shown that minimum of 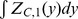, with constrains 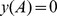 and 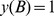, is attained when the reaction coordinate *y* equals the 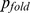 coordinate - an optimal coordinate [10]. Correspondingly, a sub-optimal coordinate with a lower value of the cut profile, has the mean square displacement which grows slower then linear with time [14]. The latter is an indication that dynamics is not diffusive and that non-Markovain memory effects are at play. Another manifestation of a sub-optimal coordinate, is that it has lower free energy barriers and thus a faster kinetics. The kinetics along the coordinate with the highest cut profile is the slowest [9], [14].

### Supervised optimization

Optimization of the reaction coordinate can be performed in a supervised or unsupervised manner. In supervised optimization a coordinate that accurately describes the dynamics of transition between two given end states (e.g., healthy and disease) is determined. Incidentally, the optimal coordinate is the probability of full recovery, i.e., of ending up in the “healthy” state rather than the “disease” state starting from a current state. The optimization is constrained by fixing the value for the coordinate for the two state 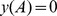 and 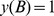 [10]. If the reaction coordinate is a weighted sum of basis functions 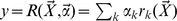, boundary conditions are given as 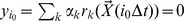 and 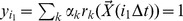 where 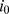 and 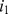 index the points which belong to A and B states, respectively. The optimal weights (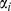) which give constrained maximum to 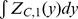 can be found analytically [10]. Here we specified the constrains in the following way, which resulted in a more flexible coordinate. Instead of assuming that each trajectory ends at either 0 or 1, we assumed that each trajectory is constrained to end with either 0 or 1 (in other words a trajectory reaches an end state on the following day). In this case the optimal weights are found by minimizing 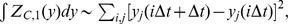 which equals
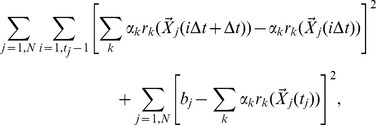
where 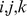 are indexes that refer to time frame, trajectory and basis function, respectively; the second term of the functional describes the boundary condition with 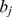 equal 0 or 1 for trajectories connected to 0 or 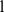, respectively. The optimal parameters are found by solving the corresponding system of linear equations 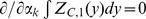. To facilitate the visual comparison with the unsupervised results 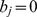 where changed to 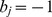, which results in a shift and change of scale of the optimal coordinate.

### Unsupervised optimization

In unsupervised optimization the determined coordinate describes the slowest relaxation mode (the second eigenvector) of the stochastic dynamics [10]. If the two states (*A* and *B*) are separated by the highest barrier, so the slowest relaxation rate corresponds to the transition dynamics between the states, the second eigenvector reaction coordinate approximates the folding probability (the probability of full recovery here) reaction coordinate in the transition state region - the most important part for the description of the transition dynamics [10], [12]. The eigenvectors can be found by minimizing 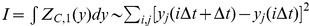 under constraint 
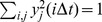
 [10]. Due to the constraint, the optimization function simplifies to the auto-correlation function. If reaction coordinate is a weighted sum of basis functions, the optimal weights can be found analytically. They are the solution of the generalized eigenvalue problem [10].

### Determination of the equilibrium free energy profile and the probability of successful outcome

The free energy profile that describes the disease dynamics cannot be determined from the patients trajectory simply by computing the cut based (or histogram) free energy profiles because the trajectories are not at equilibrium. The procedure described in Ref [13] was employed. Briefly, assuming diffusive dynamics, the equilibrium free energy profile can be computed from the steady state (non-equilibrium) probability distribution 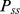 as
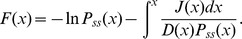
Using 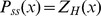, 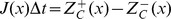 and 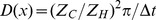, one obtains
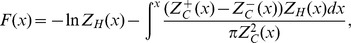
where 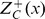 and 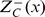 are the cut profiles that measure flux in positive and negative direction, respectively.

Note that the method for determining the optimal reaction coordinate was originally derived for equilibrium dynamics; an extension of the framework to non-equilibrium dynamics has been suggested recently [24]. Here we assume that while non-equilibrium sampling affects populations, its main effect on the optimization procedure is in altering the contribution (weight) of the different regions to the optimization functional 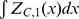 and can be neglected.

The probability of “full recovery” (the folding probability) was computed from the free energy profile as
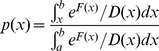
The success probability with 95% confidence interval were estimated from the trajectories by “add two successes and two failures” approach [25] as 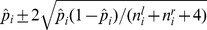, where 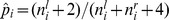 and 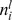 and 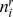 are the numbers of trajectories visiting bin 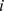 ended up in left or right half of the profile, respectively [26].

### Acquisition of NMR spectra


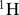
 NMR spectra were obtained for the water soluble components [27] of erythrocytes taken from 18 kidney transplant patients (up to 9 time points from pre-op to 7 days after surgery).

One-dimensional 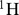 NMR spectra were measured at 499.97 MHz on a Varian Unity Inova 500 spectrometer at 20°C, using a standard PRESAT pulse sequence. For all samples a relaxation delay of ca. 9 s (three times the longest T1) was applied between scans to allow the spins to fully relax, with 256 transients collected into 16384 data points and a spectral width of 6000 Hz.

An exponential line broadening of 0.5 Hz was applied to each free induction decay (FID) and zero filling to 32768 points was carried out, followed by Fourier transformation. Phase and baseline corrections were carried out using ACD/Labs 12.0 (Advanced Chemistry Development Inc., Toronto, Canada) and chemical shifts were referenced to the lactate doublet at 1.33 ppm.

### Clinical assessment of patients

Independently of the NMR data, patients have been divided in three classes based on a clinical assessment of the patients into primary function (PF), delayed graft function (DGF) and acute rejection (AR) with and without primary function. Primary function was defined as immediate recovery of renal function. Delayed graft function was defined as the need for dialysis in the first week following transplantation. Diagnosis of acute rejection was conducted on the basis of biopsy and histological findings. Dialysis was performed on day 5 to patient 4, day 2 to patient 7, day 4 to patient 9, day 7 to patient 17 and day 6 to patient 18. All biopsies were conducted between 6 and 9 days following transplantation. Nine patients had immediate primary function, five patients had delayed graft function and four patients had acute rejection. All but one transplant were eventually successful: in addition to acute rejection, patient 14 also suffered from renal artery stenosis, and the graft was ultimately removed. The immunosuppressive regime and induction agents were the same across the cohort.

## Supporting Information

Data S1 NMR Spectra of patients.
(TGZ)
